# Adherence to endocrine therapy in early breast cancer in relation to Cytochrome P450 2D6 genotype: a comparison between pharmacy dispensation data and medical records

**DOI:** 10.1007/s10549-023-06887-2

**Published:** 2023-03-01

**Authors:** Linda Thorén, Sara Margolin, Erik Eliasson, Jonas Bergh, Jonatan D. Lindh

**Affiliations:** 1 Department of Clinical Science and Education at Södersjukhuset, Karolinska Institutet, Department of Oncology, Södersjukhuset, Stockholm, Sweden; 2 Department of Laboratory Medicine, Clinical Pharmacology, Karolinska Institutet and Medical Diagnostics, Karolinska University Hospital, Stockholm, Sweden; 3 Department of Oncology-Pathology, Karolinska Institutet and Breast Cancer Center, Cancer Theme, Karolinska University Hospital, Karolinska Comprehensive Cancer Center, Stockholm, Sweden

**Keywords:** Adjuvant endocrine treatment, Breast cancer, Adherence, Tamoxifen, CYP2D6

## Abstract

**Purpose:**

Suboptimal adherence to adjuvant endocrine treatment (AET) is an important clinical concern. A correlation between CYP2D6 activity and tamoxifen discontinuation has been described. The main aim of this study was to investigate the consistency between pharmacy dispensation data and medical records on adherence to AET.

**Methods:**

Adherence was calculated for patients with at least 4.5 years of follow up and was defined as Medical Possession Rate ≥ 80%. Subgroup analyses were performed based on menopausal status, recurrence risk and CYP2D6 activity.

**Results:**

In 86% of the 1235 included patients the consistency between the two sources of information was within 80–125%. Poor consistency, < 80%, was most frequent in the premenopausal/ high-risk group and CYP2D6 Poor Metabolizers (PMs). Among 899 patients with at least 4.5 years follow up, 72% were adherent to tamoxifen based on pharmacy dispensation data, compared with 77% as reported by medical records. When including patients who switched to aromatase inhibitors after tamoxifen, adherence increased to 82% and 88%, respectively. Adherence did not differ by menopausal status or risk for recurrence. CYP2D6 PMs had poorer adherence (54%) to tamoxifen compared to patients with the highest CYP2D6 activity (83%).

**Conclusions:**

There was a good consistency between medical records and pharmacy dispensing data on the use of AET. Adherence to AET was adequate, especially when including switch to aromatase inhibitors. Surprisingly, CYP2D6 PMs had low adherence to tamoxifen, despite a likely reduced risk of side effects according to previous data.

## Introduction

Adjuvant treatment with tamoxifen or aromatase inhibitors substantially reduces breast cancer recurrence and improves survival in patients with estrogen receptor-positive breast cancer [1–3].

A considerable proportion of patients discontinue their treatment which has a negative impact on outcome [4–7]. In a previous Swedish study as many as 50% of the patients discontinued their AET during the 5-year follow-up [8]. Suboptimal adherence to adjuvant endocrine treatment (AET) is therefore an important clinical concern. Non-adherence starts early, and the rates of discontinuation increase with each subsequent year [9–11]. Due to the heterogeneous assessment methods and definitions of adherence, the interpretation and comparison of results from previous reports is challenging. Only a few studies have used multiple methods to compare differences in measurements of adherence to AET within the same cohort [12–14].

Adverse effects are known to be major reasons for discontinuing AET [8, 9, 15]. Previous data suggest a reduced risk of side effects of tamoxifen in patients with poor CYP2D6 activity compared with those with the highest activity [16] and that CYP2D6 PMs may have better tamoxifen adherence compared to patients with higher CYP2D6 activity [17]. Concomitant use of potent CYP2D6 inhibitors might not only reduce side effects of tamoxifen, but potentially also negatively impact tamoxifen efficacy [18].

The main aim of this study was to investigate the consistency between pharmacy dispensation data and information from medical records on adherence to AET in early breast cancer. We also aimed to assess the consistency between pharmacy dispensation data and medical records on treatment with CYP2D6 inhibitors. Finally, we aimed to study if there was an association between menopausal status, CYP2D6 activity, estimated risk for recurrence and adherence to AET.

## Patients and methods

### Patients

**Fig. 1:**
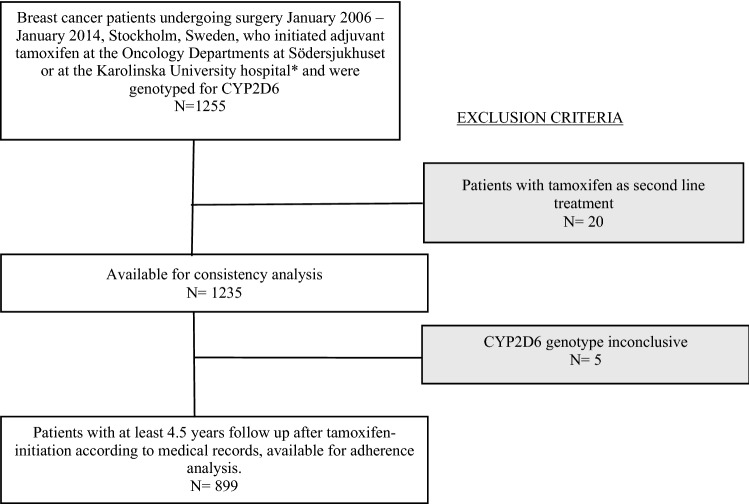
Flow chart of study participant selection and reasons for exclusion. *) When the patients were included, the Oncology Department was one unit, with two sites (Radiumhemmet and Södersjukhuset) and after 2016 two separate clinics; Departments of Oncology at Södersjukhuset and Breast Cancer Center, Cancer Theme at the Karolinska University Hospital

During several years, DNA from blood has been bio-banked from newly diagnosed breast cancer patients at Södersjukhuset and the Karolinska University Hospital, Stockholm, Sweden. By using the National Quality Registry for Breast Cancer [19] we identified 1255 patients undergoing primary breast cancer surgery January 2006—January 2014, with available DNA for CYP2D6 genotyping and who initiated adjuvant tamoxifen treatment at the Departments of Oncology, at Södersjukhuset or at the Karolinska University Hospital. Subsets of the cohort have been used in previous investigations [20, 21]. Patients were excluded if they initiated their AET with an aromatase inhibitor or a GnRH analogue alone and not tamoxifen as their first adjuvant endocrine therapy, or if their CYP2D6 genotype was inconclusive. Patients were monitored from the date of tamoxifen initiation documented in medical records, until local breast cancer recurrence, distant metastasis, contralateral cancer, death or until end of study. A detailed description of the selection of the study population is depicted in Fig. 1. Information on menopausal status, tumor characteristics, concomitant medication, breast cancer treatment, reported adherence to AET, side effects and follow-up was retrospectively obtained from medical records at the oncological departments.

Informed consent was obtained from all included patients with biobanked DNA. Approval of the study was given by the ethical review board at Karolinska Institutet (Stockholm, Sweden, Dnr 02–061, 2014/427–3 and 2016/1698–32). However, in accordance with our ethical approval, we did not approach the patients again for this specific study.

### Genotyping of CYP2D6

As described previously, analysis of CYP2D6 variant alleles was performed by TaqMan-based real time PCR assays at Diakonhjemmet Hospital in Oslo, Norway [20]. In the present study, patients were classified into predicted phenotypes; CYP2D6 poor metabolizers (PMs), CYP2D6 normal- or intermediate metabolizers (NM/IMs), or CYP2D6 ultrarapid metabolizers (UMs) in accordance with recommendations from the Clinical Pharmacogenetics Implementation Consortium and Dutch Pharmacogenetics Working Group [22]. In addition, a post hoc analysis was performed with a broader definition of CYP2D6 PMs. This was based on the observation in our previous report [20] that carriers of two reduced-function alleles, or the combination of a null-allele and a reduced-function allele, generated only very low levels of the active metabolite endoxifen and therefore may be considered as CYP2D6 PMs.

### The Swedish Prescribed Drug Register

Data from prescription renewals between January 2006 and January 2018 on AET and clinically relevant CYP2D6 inhibitors (fluoxetine, paroxetine, haloperidol, duloxetine, levomepromazine, zuclopenthixol, thioridazine, diphenhydramine, amiodarone, quinidine, terbinafine, cinacalcet and bupropion) were retrieved from the Swedish Prescribed Drug Register [23]. Sertraline was also included, as local guidelines at the time for data collection discouraged medication with this moderate CYP2D6 inhibitor together with tamoxifen.

### Definition of adherence and consistency

Medication possession ratio (MPR) estimates the proportion of prescribed days’ supply of a medication during a specified period. The lower limit of 80% is often used to define adherence to AET [14]. We therefore chose to define adherence to AET as MPR of at least 80%. As the date for the five-year follow-up could take place within a six-month time frame in clinical practice at the Oncology Departments, we chose to calculate adherence to AET for patients with at least 4.5 years of follow-up. In this study, patients were thus defined as adherent if their intake of AET covered at least 80% of at least 4.5 years of their recommended adjuvant endocrine therapy.

As it was difficult to determine whether ovarian suppressing agents had been dispensed concomitant or sequentially with tamoxifen, we focused on adherence to tamoxifen and aromatase inhibitors.

In this study consistency was used to specify the degree of concordance between the two sources of drug exposure information. Consistency was defined as dispensed amounts of AET (number of defined daily doses, DDD, divided by AET intake documented in medical records, also expressed as DDDs). Inspired by the well-established equivalence definition used in bioequivalence studies [24] and the non-inferiority exposure margins used in the short duration anti-Her-2 studies [25], we defined adequate consistency as being within the range of 80 to 125%.

## Statistical analysis

We calculated the proportion of patients with adequate consistency (80–125%), as well as those with poor consistency (< 80% or > 125%).

Predefined subgroup analyses were performed based on CYP2D6 activity, menopausal status and estimated recurrence-risk. The risk of recurrence was estimated by a compilation of prognostic factors including nodal status, tumor grade, lymph node- and Her2 status and a marker of cellular proliferation (Ki-67/S phase) [26]. Ki-67 > 20% was used to differentiate between low and high values according to the definition from the St Gallen International Expert Consensus [27]. Patients at high risk of recurrence were in this study defined as having positive lymph nodes and/or tumors with high proliferation rate (proliferation index, Ki_67_ > 20/S phase > 10%) and/or grade III and/or Her2 amplification and/or having received chemotherapy. Fisher's exact test was used to compare adherence frequencies between groups, and 95% CIs were calculated. Information on exposure to a CYP2D6 inhibitor at least once during the follow-up was compared between the prescribed drug register and medical records.

Statistical analyses were performed using R 3.6.1 [R Core Team (2019). R: A language and environment for statistical computing. R Foundation for Statistical Computing, Vienna, Austria. URL https://www.R-project.org/].

## Results

In all, 1235 women were included in the study (Fig. 1). The majority (78%) of the patients received tamoxifen as their only adjuvant endocrine treatment. Fourteen percent of the patients switched their adjuvant endocrine therapy from tamoxifen to an aromatase inhibitor. Forty one percent of the patients were premenopausal. Forty percent of the patients had a high estimated risk of recurrence. Seven percent were CYP2D6 PMs, 90% were NM/IMs and 3% were UMs. The groups of CYP2D6 UMs and PMs were well balanced as for age, menopausal status and estimated risk of recurrence (data not shown). Patient baseline characteristics are summarized in Table 1.

**Table 1:** Baseline characteristics of study participants

Characteristic	Value
*N*	1235
Age at breast cancer diagnosis, median, range, (IQR)	57, 21–89, (47–66)
Premenopausal, *n*, (%) Postmenopausal, *n*, (%) Perimenopausal/uncertain menopausal status, (%)	500, (40.5) 711, (57.6) 24, (1,9)
Age at menopause, median, (IQR)	50 (48–52)
ER positive, *n*, (%) ^a^ ER negative, *n*, (%)^b^ Missing, *n*, (%)	1227, (99.4) 4, (0.3) 4, (0.3)
PR positive, *n*, (%) PR negative, *n*, (%) Missing, *n*, (%)	1052, (85.2) 174, (14.1) 9, (0.7)
Tumor size, < 20 mm,* n, (%)* 21–50 mm,* n, (%)* > 50 mm,* n, (%)* Missing,* n, (%)*	880, (71.2) 280, (22.7) 70, (5.7) 5, (0.4)
Tumor grade I*, n, *(%*)* II*, n, *(%) III,* n, *(%) Missing*, n, *(%)	403, (32.6) 613, (49.6) 195, (15.8) 24, (1.9)
Lymph node status N_0_, *n*, (%) N+ *, n, *(%) Missing*, n*, (%)	997, (80.7) 231, (18.7) 7, (0.6)
Ki_67_ < 20/S phase < 10%*, n*, (%) Ki_67_ > 20/S phase > 10%*, n*, (%) Missing*, n*, (%)	901, (73.0) 298, (24.1) 36, (2.9)
Her2 positive, *n*, (%) Missing*, n, *(%)	62, (5.0) 16, (1.3)
“High risk patients”^c^, *n*, (%) Premenopausal, *n*, (%) Postmenopausal, *n*, (%)	494, (40.0) 343, (68.6) 142, (20.0)
Chemotherapy All patients, *n*, (%) Premenopausal patients, *n*, (%) Postmenopausal patients, *n*, (%)	330, (26.7) 291, (58.1) 34, (4.8)
Endocrine treatment full cohort Tamoxifen only, *n*, (%) Tamoxifen and goserelin, *n*, (%) Tamoxifen and aromatase inhibitor, *n*, (%) Tamoxifen, goserelin and aromatase inhibitor, *n*, (%)	961, (77.8) 119, (9.6) 169, (13.7) 14, (1.1)
Endocrine treatment premenopausal patients Tamoxifen only, *n*, (%) Tamoxifen and goserelin, *n*, (%) Tamoxifen and aromatase inhibitor, *n*, (%) Tamoxifen, goserelin and aromatase inhibitor, *n*, (%)	344, (68.8) 119, (23.8) 51, (10.2) 14, (2.8)
Endocrine treatment postmenopausal patients Tamoxifen only, *n*, (%) Tamoxifen and goserelin, *n*, (%) Tamoxifen and aromatase inhibitor, *n*, (%) Tamoxifen, goserelin and aromatase inhibitor, *n*, (%)	603, (84.8) – 108, (15.2) –
Follow up time, months, median, (IQR)	77.6 (55.2–101.8)
CYP2D6- activity according to CPIC’s guidelines[22] CYP2D6-Poor Metabolizers (PM),* n, (%)* CYP2D6-Normal-/Intermediate Metabolizers, *n, (%)* CYP2D6-Ultrarapid Metabolizers (UM), *n, %*	90, (7.2) 1105, (89.5) 35, (2.8)

Percentages may not add up to 100 due to rounding a) Tumors were considered Estrogen Receptor (ER) positive and Progesterone Receptor (PR) positive if ≥  **>**10% of the cells stained positive for the receptor by immunohistochemistry.b) 3 patients were ER-negative, but PR positive and thus defined as Hormone Receptor (HR) positive. The 4 patients where ER-status was missing were treated as HR-positive. c) Patients were considered at “high risk” for recurrence if tumor grade III and/or Ki67 > 20/S phase > 10% and/or N+ , and / or Her2-positive and/or treated with chemotherapy

**Fig. 2:**
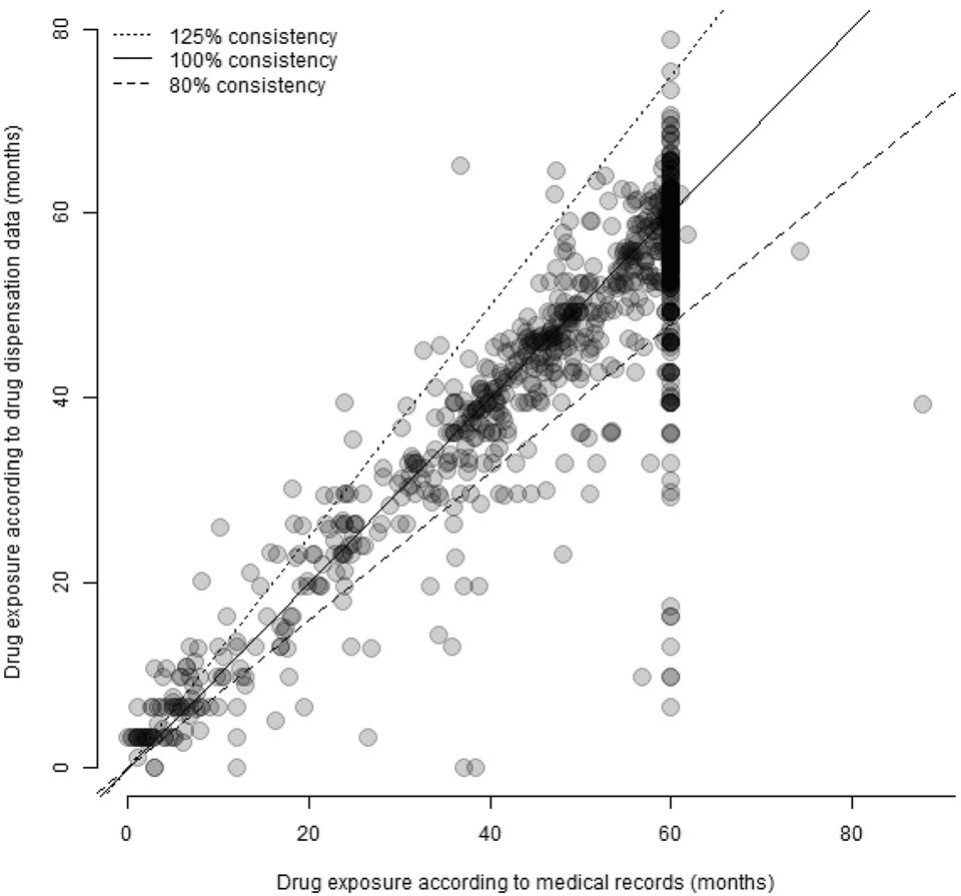
Consistency between drug exposure to AET according to drug dispensation data and medical records

In 84% of the patients there was adequate consistency between the two sources of information on exposure to adjuvant tamoxifen. When including switch to an aromatase inhibitor the proportion of patients with adequate consistency increased to 86%. Poor consistency, < 80% (i.e. fewer dispensed doses than the total use documented in the medical records) was seen in 9% of all patients for all AET and poor consistency > 125%, (i.e. more doses dispensed in the pharmacies than the total use documented in the medical records) was found in 5% of all patients for all AET. Poor consistency < 80% was most frequent in premenopausal-/high-risk patients and > 125% was most common in postmenopausal-/low risk patients. CYP2D6 PMs were overrepresented in both groups (Fig. 2, Table 2). Similar results were found in the post hoc analysis based on an expanded definition of CYP2D6 PMs (see Methods, data not shown).

**Table 2:** Consistency between dispensed doses of AET and AET intake documented in medical records during follow-up, in relation to menopausal status, patients’ risk for recurrence and CYP2D6 activity

Tamoxifen	Poor consistency, dispensed doses/intake in medical records < 80%		Adequate consistency, dispensed doses/intake in medical records 80–125%		Poor consistency, dispensed doses/intake in medical records > 125%	
	*n*, (%)	CI	*n*, (%)	CI	*n*, (%)	CI
All patients (*n* = 1235)	107, (8.7%)	0.07, 0.10	1043, (84.4%)	0.82, 0.86	85, (6.9%)	0.06, 0.08
Premenopausal patients (*n* = 500)	61, (12.2%)	0.10, 0.15	421, (84.2%)	0.80, 0.87	18, (3.6%)	0.02, 0.06
Postmenopausal Patients (*n* = 711)	44, (6.2%)	0.05, 0.08	603, (84.8%)	0.82, 0.87	64, (9.0%)	0.07, 0.11
High risk patients^a^ (*n* = 494)	56, (11.3%)	0.9, 0.15	418, (84.6%)	0.81, 0.88	20, (4.0%)	0.03, 0.06
Low risk patients (*n* = 741)	51, (6.9%)	0.05, 0.09	625, (84.3%)	0.81, 0.87	65, (8.8%)	0.07, 0.11
CYP2D6 PM^b^ (*n* = 90)	12, (13.3%)	0.07, 0.23	67, (74.4%)	0.64, 0.83	11, (12.2%)	0.07, 0.21
CYP2D6 NM/IM^b^ (*n* = 1105)	91, (8.2%)	0.67, 0.10	942, (85.2%)	0.83, 0.87	72, (6.5%)	0.05, 0.81
CYP2D6 UM^b^ (*n* = 35)	2, (5.7%)	0.01, 0.21	31, (88.6%)	0.72, 0.96	2, (5.7%)	0.01, 0.21

All oral AET	Poor consistency, dispensed doses/intake in medical records < 80%		Adequate consistency, dispensed doses/intake in medical records 80–125%		Poor consistency, dispensed doses/intake in medical records > 125%	
	*n*, (%)	CI	*n*, (%)	CI	*n*, (%)	CI
All patients (*n* = 1235)	111, (8.9%)	0.07, 0.11	1062, (86.0%)	0.84, 0.88	62, (5.0%)	0.04, 0.06
Premenopausal patients (*n* = 500)	65, (13.0%)	0.10, 0.16	420, (84.0%)	0.80, 0.87	15, (3.0%)	0.02, 0.05
Postmenopausal Patients (*n* = 711)	44, (6.2%)	0.05, 0.08	621, (87.3%)	0.85, 0.90	46, (6.5%)	0.05, 0.09
High risk patients^a^ (*n* = 494)	61, (12.3%)	0.10, 0.16	417, (84.4%)	0.81, 0.87	16, (3.2%)	0.02, 0.05
Low risk patients (*n* = 741)	50, (6.7%)	0.05, 0.09	645, (87.0%)	0.84, 0.89	46, (6.2%)	0.05, 0.08
CYP2D6 PM^b^ (*n* = 90)	14, (15.5%)	0.09, 0.25	70, (77.8%)	0.68, 0.86	6, (6.7%)	0.03, 0.14
CYP2D6 NM/IM^b^ (*n* = 1105)	94, (8.5%)	0.70, 0.10	957, (86.6%)	0.84, 0.89	54, (4.9%)	0.04, 0.06
CYP2D6 UM^b^ (*n* = 35)	2, (5.7%)	0.01, 0.21	31, (88.6%)	0.72, 0.96	2, (5.7%)	0.01, 0.21

Consistency between the two sources of information on adherence was calculated by dividing dispensed doses of AET by AET intake documented in medical records. The proportion of patients with adequate consistency (80–125%), as well as those with poor consistency (< 80% and > 125%) with 95% confidence intervals (CI), was computed

^a^A high risk of recurrence was defined as having positive lymph nodes and/or had tumors with high proliferation rate (Ki_67_ > 20/S phase > 10%) and/or grade III and/or Her2 amplification and/or had received chemotherapy

^b^Patients were classified into predicted CYP2D6 phenotypes; CYP2D6 poor metabolizers (PMs) CYP2D6 normal- /intermediate metabolizers (NM/IMs), or CYP2D6 ultrarapid metabolizers (UMs) according to consensus recommendations from the Clinical Pharmacogenetics Implementation Consortium and Dutch Pharmacogenetics Working Group[22]

**Fig. 3:**
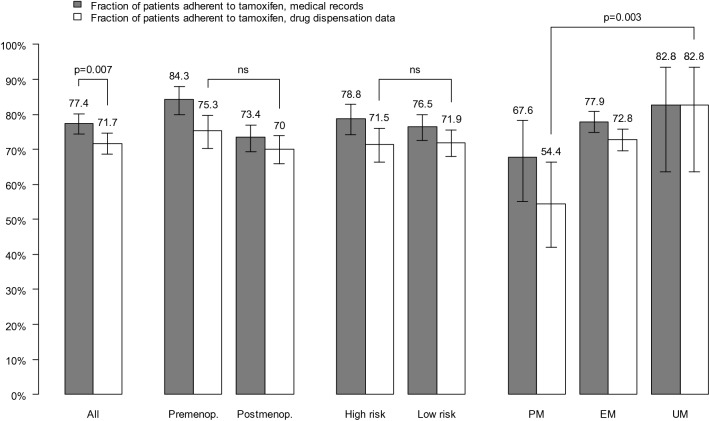
Fraction of patients adherent to tamoxifen according to medical records and pharmacy dispensation data. Adherence to tamoxifen was defined as a medical possession rate (MPR) of at least 80% over a follow-up period of 4.5 to 5 years

**Fig. 4:**
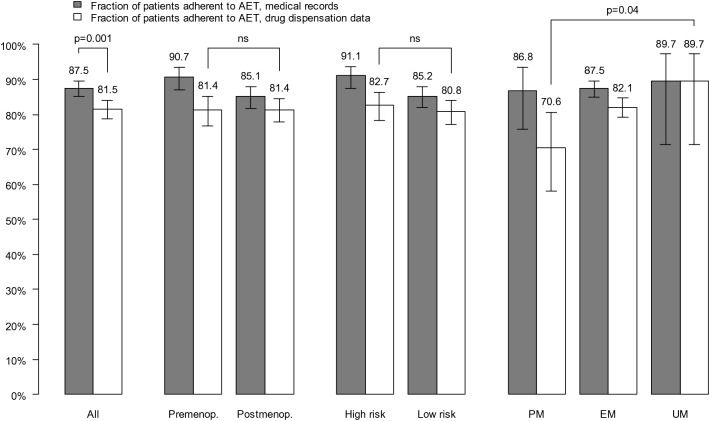
Fraction of patients adherent to AET according to medical records and pharmacy dispensation data. Adherence to AET was defined as a MPR of at least 80% over a follow-up period of 4.5 to 5 years

Adherence to tamoxifen was 72% based on pharmacy dispensation data, compared to 77% reported by medical records. When including switch to aromatase inhibitors adherence increased to 82% and 88%, respectively. Adherence did not differ by menopausal status or risk for recurrence. CYP2D6 PMs had poorer adherence (54%) not only to tamoxifen, but also to aromatase inhibitors (71%) compared to patients with normal CYP2D6 activity (73% and 82%, respectively) as well as those with the highest CYP2D6 activity (83% and 90%, respectively) based on pharmacy dispensation data (Figs. 3 and 4). The effect of CYP2D6 phenotype on adherence remained significant for tamoxifen (but not for AET in general) in the post hoc analysis using an expanded definition of CYP2D6 PM. More specifically, CYP2D6 PM still had poorer adherence (62%) to tamoxifen, compared to patients with normal CYP2D6 activity (73%) and CYP2D6 UM (83%).

According to medical records, 52 patients were treated with CYP2D6 inhibitors. 73 additional patients had been dispensed CYP2D6 inhibiting medication. Consequently, in 58% of the patients with dispensed CYP2D6 inhibitors, care givers at the oncological departments were either unaware of or did not document the CYP2D6-inhibiting medication. In contrast, 6 patients who based on medical records had received CYP2D6 inhibitors, never filled their prescription.

## Discussion

In this cohort of tamoxifen-treated early breast cancer there was a good agreement, consistency, between medical records and dispensing data on the use of AET. The proportion of patients with adequate adherence to adjuvant endocrine treatment was satisfactory, 82% when including switch to aromatase inhibitors. Surprisingly, CYP2D6 PMs had low adherence to tamoxifen.

Although adherence is important for all studies on AET, there is to date no gold standard for adherence evaluation, nor for comparing adherence to AET by different methods [28]. Dispensing data is usually regarded as the most accurate method for drug exposure information as it is not susceptible to recall bias. Previous studies have revealed that patients tend to underreport treatment discontinuation [29, 30]. Information from medical records might thus overestimate adherence. This was also reported by Font et al., where adherence to AET was calculated up to five years from the date of the first prescription refill and deemed satisfactory if MPR was 80–110%. Adherence at 5 years based on medical records was higher, 95%, than prescription refill data, 75% [12]. The discrepancies between these two sources of information on adherence was slightly less pronounced in the present study. In previous research [12] the kappa statistic has been used as a measure of agreement between different sources of adherence data. Due to the symmetric nature of the kappa measure, where no data source is formally given greater credence than another, we instead chose an asymmetric approach addressing how well the medical records performed, with the prescribed drug register as “gold standard”. However, to enable across-study comparisons, we have analyzed the agreement between 5-year adherence estimates from the two data sources by means of Cohen's kappa in a post hoc analysis. For the full cohort, there was a near perfect agreement regarding adherence to tamoxifen (kappa 0.82, 95% CI 0.78; 0.86), and a substantial agreement for AET (kappa 0.72, 95% CI 0.66; 0.78). As expected from the adherence results presented above, the lowest agreement was seen among CYP2D6 PMs (kappa 0.73 for tamoxifen adherence, 0.54 for AET adherence) and premenopausal women (kappa 0.69 for tamoxifen adherence, 0.57 for AET adherence). In the small group of CYP2D6 UMs there was a perfect agreement regarding both tamoxifen and AET adherence (kappa 1, with all 29 individuals receiving identical classifications from the two data sources). In the aforementioned study, concordance among the three used methods to assess adherence was substantially lower (kappa 0.02 to 0.27) [12].

Fewer doses actually dispensed than reported by the patient in medical records, was more frequently seen in premenopausal-/“high risk” patients. Side effects to AET may considerably affect patients’ quality of life [31]. It has been suggested that both adverse effects and fertility concerns contribute to lower adherence to tamoxifen in premenopausal women [32]. In this study, the majority of the patients defined at high risk of recurrence were premenopausal. Younger patients, especially those at higher risk of recurrence, may be more reluctant to admit that they do not follow the recommended treatment duration of AET.

More doses dispensed than the total use of tamoxifen documented in the medical records was concordantly more common in postmenopausal-/low-risk patients. Some of these patients had likely also been prescribed endocrine treatment at another caregiver, whose medical records were not available at the time of data collection. In patients switching AET, refill data might also overestimate adherence due to overlap of prescribed medication. Some patients filled multiple prescriptions for AET. In addition, a few patients were prescribed aromatase inhibitors during in vitro stimulated fertilization, after cancer diagnosis.

The range of five-year adherence to AET in previous studies is wide; from around 30 to 90% [9, 14]. Some of this variability may be explained by the use of different measures of adherence. In our present study, adherence to adjuvant tamoxifen was considered adequate, 72%, and increased to 82%, when individual switches to aromatase inhibitors were also considered. In another recent Swedish register study, it was concluded that only 50% of patients continued AET treatment as planned during the 5-year follow-up [8]. However, in that study a lower threshold for discontinuation was used. The results of the present investigation based on MPR of 80% are more in line with other recent work that applied a similar definition of AET adherence [33–35]. Previous studies have reported younger age as a factor contributing to reduced adherence [9, 14], while being diagnosed with an advanced cancer has been suggested to motivate patients to remain adherent to AET [35]. In contrast, we did not find that adherence differed by menopausal status or risk for recurrence. Our results highlight the importance of more effective communication on the benefits of AET and better management of adverse effects, especially for younger patients with a higher risk for recurrence. Uniform definitions and measurements of adherence in future studies would facilitate both comparisons of data and pinpointing where targeted interventions are most needed.

The exposure to CYP2D6 inhibitors was higher according to drug dispensation data (10%) compared to medical records (4%). The number of patients who might be at risk of reduced tamoxifen efficacy due to concomitant CYP2D6 inhibitor treatment could be larger than anticipated and an increased awareness of such drug–drug interactions is relevant.

Previous data suggest a reduced risk of side effects to tamoxifen in patients with poor CYP2D6 activity compared to those with the highest activity [16] and that treatment discontinuation during the initial 4 months is lower among CYP2D6 PMs than other genotypes [17]. In another study, CYP2D6 UMs were three times more likely than CYP2D6 NMs to discontinue tamoxifen in the early phase, proposedly linked to tamoxifen adverse drug reactions as evidence was found that CYP2D6 UM patients consumed more of corresponding symptom relief pharmaceuticals [36]. In the present study, 126 patients switched from tamoxifen to an aromatase inhibitor due to side effects during the first five years, according to medical records. Of these 108 (86%) were CYP2D6 NM/IMs, 14 (11%) CYP2D6 PMs and 4 (3%) CYP2D6 UMs, which largely reflects the distribution of CYP2D6-activity in the whole study population. Surprisingly, CYP2D6 PMs had both poorer long-term adherence (54%) to tamoxifen treatment compared to patients with the highest CYP2D6 activity (83%) as well as the lowest consistency between reported intake and pharmacy data on prescription refills, compared to other subgroups. There is no simple explanation for these observations. It is possible that there was a bias concerning other factors of importance for adherence to AET, such as comorbidity and use of other symptom relieving drugs [8, 35, 37] that we do not have information on, between the different CYP2D6 activity groups. The side effects that CYP2D6 UMs experienced might also have prompted increased contact with health care providers motivating them to continue their treatment, while for CYP2D6 PMs the possible lack of side effects might have rendered patients skeptical of the treatment´s benefits. As patients carrying a poor CYP2D6 metabolizer genotype are thought to benefit less from tamoxifen treatment [38], poorer adherence among CYP2D6 PMs might however not further affect the prognosis for this group. Further studies are needed to disentangle the effect of CYP2D6 activity on adherence to tamoxifen.

Strengths of this study include the prospective collection of unselected breast cancer patients, with adjuvant treatment reflecting modern clinical practice. As no method used to assess adherence guarantees that a patient takes the medication as recommended, the true adherence to AET may be lower than reported. Other important limitations include the retrospective collection of information on adherence and that medical records from all possible caregivers were not accessible. Different health care providers might not only vary in how they communicate with patients to motivate them to continue their treatment [39], but also how they evaluate and document adherence to AET in medical records. This highlights the importance of more conform criteria for treating physicians assessing and documenting data on adherence. As many treating physicians/nurses were involved in the follow up of the patients in this study, we however believe that the sum of documented side effects in this study is reasonably average. In other health care systems dispensing databases and routines for follow up of patients might differ, which might affect the external validity of our data. In patients switching AET, pharmacy dispensation data might also overestimate adherence due to a possible overlap of prescribed medication. DNA was bio-banked from around 60% of the patients during the time span when the study patients were diagnosed with breast cancer. Although it is unlikely that adherence data would largely differ in patients without available DNA, a possible selection bias cannot completely be excluded.

In summary, in this cohort of tamoxifen-treated early breast cancer there was a good agreement, consistency, between medical records and dispensing data on the use of AET.

This might be valuable information for future studies on AET, as collecting drug dispensation data on adherence might not always be feasible. Improved understanding of factors affecting adherence to tamoxifen is crucial, so that targeted interventions to improve adherence can be implemented in the clinic. Although adherence to AET overall was adequate, tailored and accessible interventions are needed to ensure increased adherence to AET, especially in younger patients with a higher estimated risk of recurrence. Surprisingly, CYP2D6 PMs had markedly lower adherence to tamoxifen, despite a reduced risk of side effects based on previous studies. Our findings indicate that more work is needed to clarify the impact of CYP2D6 activity on adherence to tamoxifen, and whether individualized dosing of tamoxifen based on CYP2D6 genotype, aiming at improving patients’ quality of life and adherence, might further decrease the risk of recurrence and breast cancer mortality.

## Data Availability

The data that support the findings of this study are available from the corresponding author upon reasonable request.

## Code availability

The code that support the analyses of this study are available from the corresponding author upon reasonable request.
